# Multiple breath washout lung function reveals ventilation inhomogeneity unresponsive to mechanical assisted cough in patients with neuromuscular disease

**DOI:** 10.1186/s12890-022-02012-z

**Published:** 2022-06-04

**Authors:** Mathis Steindor, Anna Pichler, Laura Heitschmidt, Eva Pitsikoulis, Alexandra Kavvalou, Eser Orhan, Margerete Olivier, Florian Stehling

**Affiliations:** 1 Pediatric Pulmonology and Sleep Medicine, Department of Paediatrics III, University Children’s Hospital Essen, University of Duisburg-Essen, Hufelandstr. 55, 45122 Essen, Germany; 2 Pediatric Research Network Essen, Essen, Germany

**Keywords:** Chronic respiratory insufficiency, Pediatric pulmonology, Airway clearance, Lung function

## Abstract

**Background:**

Respiratory involvement defines the clinical outcome of neuromuscular diseases (NMD). The lung clearance index (LCI) is a marker of lung ventilation inhomogeneity and indicates small airway disease. It is determined by mulitple breath washout lung function (MBW). The merit of LCI is undisputed for primary lung diseases like cystic fibrosis, but its role in NMD is unclear.

**Methods:**

We investigated the role of MBW in patients with NMD and the effect of two different tracer gases and cough assist devices on the LCI. Patients and controls performed MBW with nitrogen (N_2_) and sulfur hexafluoride (SF_6_), whereas the latter analysis was repeated after the use of a cough assist device in the NMD group. LCI was compared to forced vital capacity (FVC) and peak cough flow (PCF).

**Results:**

24 NMD patients (12 Duchenne Muscular Dystrophy, 8 Spinal Muscular Atrophy, 4 other NMDs) and 15 healthy controls were enrolled. In the NMD group, overall LCI N_2_ was higher than LCI SF_6_ (9.67 ± 1.56 vs. 8.71 ± 1.47; mean ± SD; *p* < 0.033). In controls, LCI N_2_ did not differ significantly from LCI SF_6_ (7.03 ± 0.37 vs. 7.05 ± 0.67; *p* = 0.882). Both LCI N_2_ and LCI SF_6_ were significantly higher in NMD patients as in controls (9.67 ± 1.56 vs. 7.03 ± 0.37, *p* < 0.001, and 8.71 ± 1.478.65 vs. 7.05 ± 0.67, *p* < 0.001). In the NMD group, both LCI N_2_ and LCI SF_6_ showed a negative correlation to FVC (r = − 0.525; *p* = 0.008 and r = − 0.526; *p* = 0.008, respectively) and PCF (r = − 0.590; *p* = 0.002 and r = − 0.641; *p* = 0.001, respectively). LCI N_2_ and LCI SF_6_ correlated well in the NMD group. LCI SF_6_ did not change significantly after the use of the cough assist in NMD patients (n = 22; 8.65 ± 1.52 pre vs. 8.79 ± 2.03 post, *p* = 0.667).

**Conclusion:**

Lung involvement of patients with neuromuscular diseases goes beyond weakness of respiratory muscles. MBW with both N_2_ and SF_6_ is suitable to detect ventilation inhomogeneity in NMD patients with respiratory impairment. Cough assist devices with low to moderate pressure levels do not immediately improve the LCI.

**Supplementary Information:**

The online version contains supplementary material available at 10.1186/s12890-022-02012-z.

## Introduction

After being widely neglected over decades following its introduction in 1952 [1], multiple-breath-washout (MBW) lung function techniques have been rediscovered in recent years. The lung washout of tracer gases (usually nitrogen, N_2_, or Sulphur hexafluoride, SF_6_) to fractions (usually 5 or 2.5%) of its initial concentration is utilized to determine the lung clearance index (LCI), which is a marker of ventilation inhomogeneity, a hallmark of small airway disease [2]. Meanwhile, MBW is well established to characterize lung involvement in cystic fibrosis [3], but its merit for other diseases needs further investigation, for example for neuromuscular diseases (NMD). The spectrum of NMD comprises mostly inborn disorders of muscular function with classical examples being Duchenne Muscular Dystrophy (DMD) and Spinal Muscular Atrophy (SMA). Increasing weakness of respiratory muscles, concomitant chest wall stiffness and skeletal deformities (especially scoliosis) often cause a “complex restrictive pattern” in pulmonary function testing in NMD patients, characterized by a disproportionally reduced vital capacity relative to total lung capacity [4]. The progressive loss of vital capacity leads to impaired ventilation and cough insufficiency. Recurrent infections, reduced mucociliary clearance and hampered ventilation mechanics further affect the respiratory system in the course of the diseases, leading to a secondary lung disease exceeding the mere skeletomuscular ventilatory insufficiency [5–7]. Conventional pulmonary function testing is insufficient to distinguish between thoracic restriction and structural lung disease. In DMD patients, decreasing vital capacity strongly correlates with increasing LCI N_2_, underlining the value of MBW to characterize NMD associated airway disease [5].

To treat lung involvement of NMD patients, cough assist devices are widely used for airway clearance and lung recruitment. They lead to a measurable short-lasting increase of vital capacity and decelerate its decline in the course of several NMD [8–12]. Their effect on lung ventilation homogeneity has not been investigated. Additionally, the choice of tracer gas influences the LCI, with LCI N_2_ values usually being higher than LCI SF_6_ values in healthy and non-NMD cohorts [13–15]. The impact of the tracer gas might be different in unique ventilation mechanics of NMD lungs. In this study, we investigated MBW lung function for patients with different NMD and the influence of different tracer gases and cough assist devices on the LCI in this cohort.

## Methods

Inclusion criteria for NMD patients were established diagnosis of NMD with respiratory muscle involvement (diagnosed clinically and/or by pulmonary function testing). NMD patients and age matched healthy controls performed spirometry (Easy One, NDD Medical Technologies, Switzerland), peak cough flow measurement (PCF; Pocket Peak, nSpire Health Ltd., UK) and MBW. The best of three consecutive expiratory forced vital capacity (FVC) and PCF manoeuvres values were noted. MBW was performed sequentially with the tracer gases N_2_ and SF_6_ on an Exhalyzer D (Ecomedics, Switzerland) using the Spiroware Version 3.2.1 software for all study subjects and controls and LCI was calculated for the 2.5% stopping point (LCI 2.5) for both tracer gases. For the NMD group, MBW and LCI SF_6_ calculation was performed again after the use of the subject’s personal cough assist device with the personal settings or, if the patient was not equipped with a personal device, with a default device ad hoc titrated to the patients’ requirements. Cough assist use in the study comprised three consecutive cycles of three cough manoeuvres (3 × 3) over a period of 5 min. Patients unfamiliar with the use of cough assist devices received initial instructions and training before the 3 × 3 manoeuvres. All cough assist manoeuvres and cough assist titrations were performed and supervised by a respiratory nurse. LCI N_2_ was compared to LCI SF_6_ within each group and both analyses were compared between NMD patients and controls, LCI SF_6_ measurements before and after the use of the cough assist were compared for the NMD study group. Correlation analyses of LCI, FVC and PCF were performed. All MBW measurements and interpretations were performed according to ERS/ATS quality standards under the supervision of trained staff [16]. For each subject and analysis, two technically acceptable sequential MBW measurements with a coefficient of variation (CV) < 5% or three technically acceptable sequential MBW measurements with a CV < 10% were considered eligible for inclusion.

Statistical analyses were performed with SPSS Version 27 (IBM, USA) and Graphpad Prism Version 9.2.0 (GraphPad, USA). Kolmogorov–Smirnov-Lilliefors- and Shapiro–Wilk-test were used to determine normal distribution of non-descriptive study datasets. Unpaired and paired t-test were used for comparison between two groups and pre-/post-analyses, respectively. The relation between study parameters was determined using Pearson’s correlation and linear regression. Bland–Altman-plots were used to compare LCI results with different tracer gases to estimate the risk of bias.

## Results

**Fig. 1:**
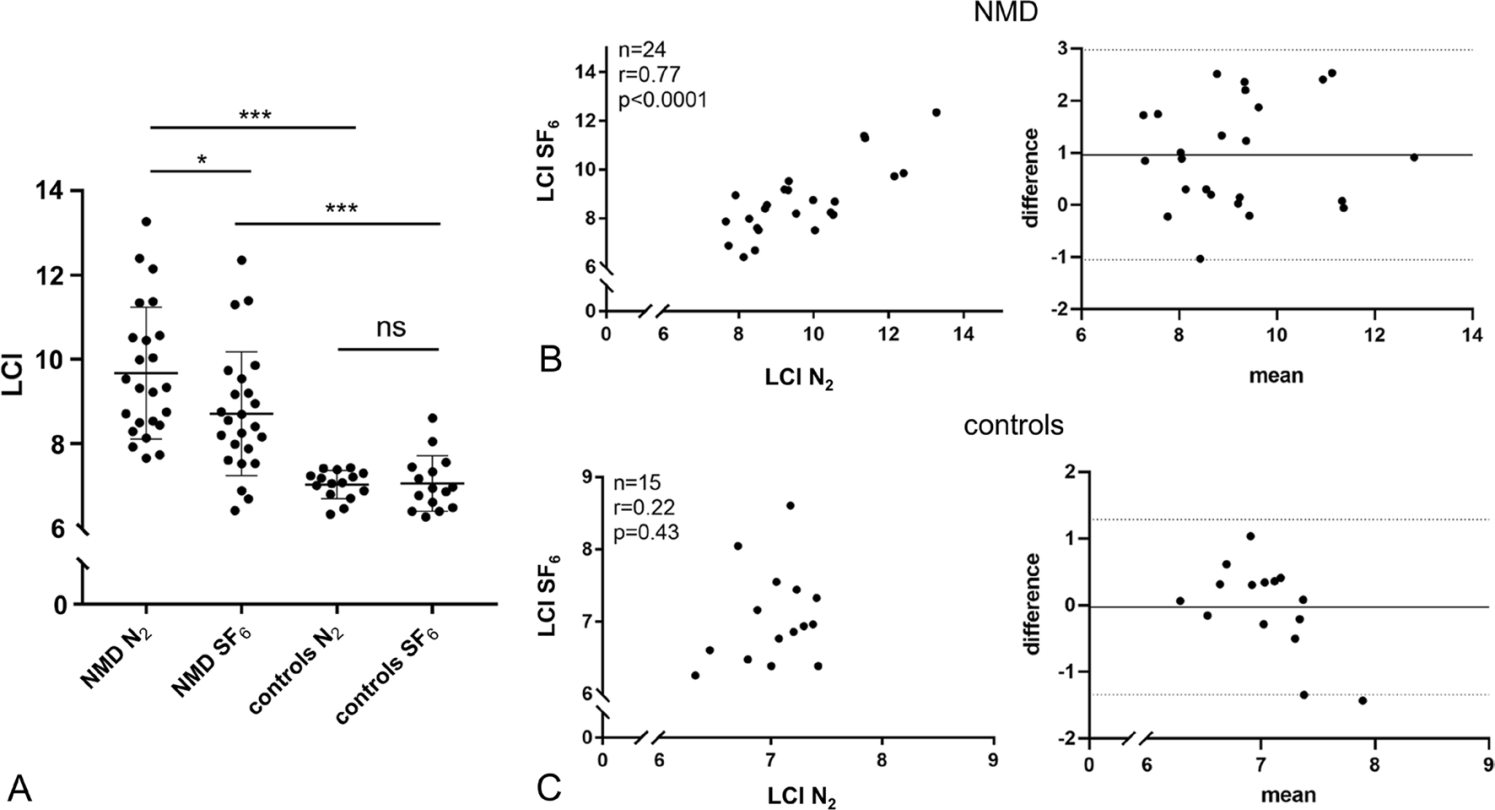
The effect of tracer gases on the LCI in NMD patients and controls **A** Scatter plot of LCI values for NMD patients and controls for the tracer gases N_2_ and SF_6_, mean and standard deviation are indicated **B** Pearson correlation (left panel) and Bland–Altman-diagram of LCI N_2_ and LCI SF_6_ (right panel) in NMD patients **C** Pearson correlation (left panel) and Bland–Altman-diagram of LCI N_2_ and LCI SF_6_ (right panel) in healthy controls

**Fig. 2:**
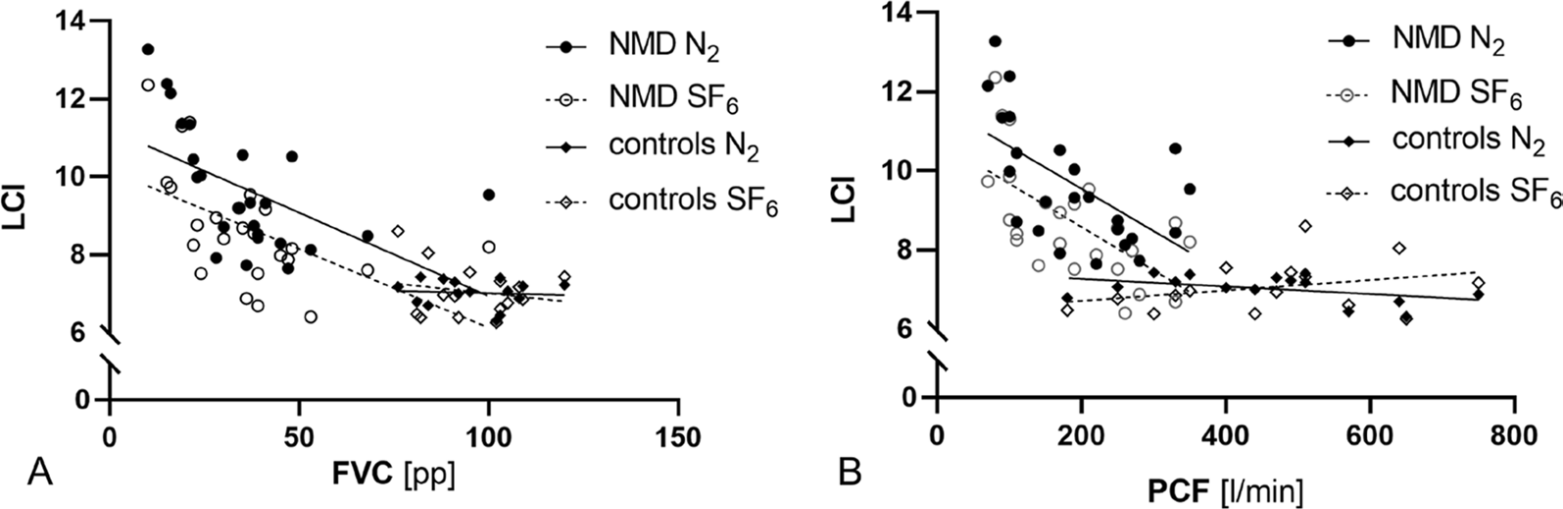
Linear regression analysis of LCI N_2_ and LCI SF_6_ in relation to forced vital capacity (FVC, Panel A) and peak cough flow (PCF, Panel B) in NMD patients and controls

**Fig. 3:**
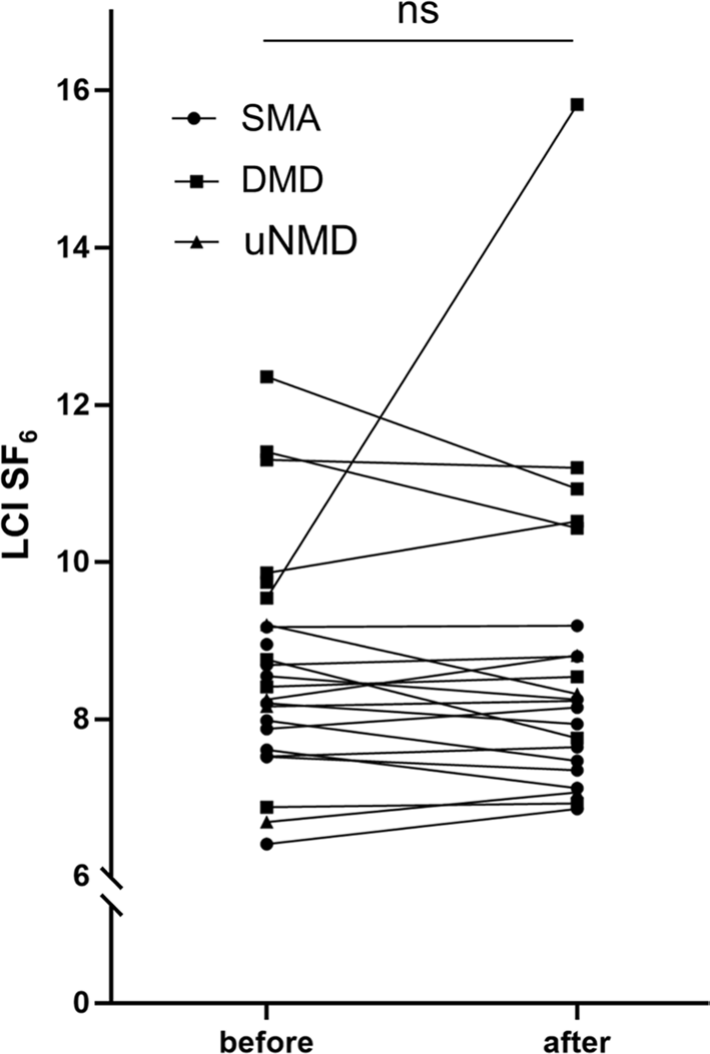
LCI SF_6_ before and after the use of a cough assist device. DMD Duchenne Muscular Dystrophy SMA Spinal Muscular Atrophie uNMD uncategorized neuromuscular disease

We enrolled 27 patients and 15 age matched healthy controls in the study. Three extremely weak NMD patients failed to perform valid MBW measurements due to persistent mouth leaks (two female SMA patients age 13 and 17 years and one female patient with unspecified NMD aged 5 years) and were excluded. Of the 24 NMD patients finally enrolled, 12 had DMD, 8 had SMA and 4 had other NMDs. Most patients showed severe restrictive patterns on spirometry and had measurable cough insufficiency (patient characteristics and study datasets are given in Table 1). All patients but three out of 24 (87.5%) were supplied with a cough assist device before enrolment in the study. The same proportion of patients had established nocturnal non-invasive ventilation (Table 1). Cough assist inspiratory pressure levels were 24.8 ± 4.0cmH_2_0 (mean ± SD), expiratory pressure levels were − 23.3 ± 5cmH_2_0. After uniform technical synchronization of MBW analyses, three SMA patients showed CV values slightly above the 5%-threshold for their two consecutive LCI N_2_ measurements (CV 6.7, 7.2 and 8.7%, respectively), one of those showed an elevated CV for the LCI SF_6_ before the use of the cough assist as well (7.1%). Decision was made to include the measurement in the analyses. Two NMD (one DMD and one SMA) patients refused to perform MBW after the use of their cough assist due to (muscular) exhaustion. In the NMD group, overall LCI N_2_ was significantly higher than LCI SF_6_ (9.67 ± 1.56 vs. 8.71 ± 1.47; mean ± SD; *p* < 0.033) (Fig. 1, Panel A). In the control group, LCI N_2_ did not differ significantly from LCI SF_6_ (7.03 ± 0.37 vs. 7.05 ± 0.67; *p* = 0.882) (Fig. 1, Panel A). In the NMD group, both LCI N_2_ and LCI SF_6_ showed a negative correlation to FVC (r = − 0.525; *p* = 0.008 and r = − 0.526; *p* = 0.008, respectively) and PCF (r = − 0.590; *p* = 0.002 and r = − 0.641; *p* = 0.001, respectively) (Fig. 2), indicating a higher LCI for both tracer gases in patients with lower FVC and PCF. Both the LCI N_2_ and the LCI SF_6_ were significantly higher in the NMD group as compared to the controls (9.67 ± 1.56 vs. 7.03 ± 0.37, *p* < 0.001, and 8.71 ± 1.47 vs. 7.05 ± 0.67, *p* < 0.001) (Fig. 1, Panel A). Notably, every subgroup of NMD (i.e. DMD, SMA and others) has significantly higher LCI N_2_ and LCI SF_6_ as compared to controls (Additional file 1: Figure S1). LCI N_2_ and the LCI SF_6_ correlated very well in the NMD group but not in the control group with bias being + 0.96 for NMD patients and -0.03 for controls according to Bland–Altman analysis (Fig. 1, Panel B/C). LCI SF_6_ did not change significantly after the use of the cough assist in NMD patients (n = 22; 8.71 ± 1.52 pre vs. 8.79 ± 2.03 post, *p* = 0.667) (Fig. 3, Panel A). One patient with DMD had a marked increase of LCI SF_6_ after the use of the cough assist (9.54 vs. 15.82), widely exceeding the changes seen in all other patients. Notably, exclusion of this patient from pre-/post-analyses did not change the statistical outcome (n = 21; 8.61 ± 1.54 pre vs. 8.45 ± 1.32 post, *p* = 0.214) (Fig. 3).

**Table 1:** Patient characteristics and study datasets for each study group. NMD neuromuscular disease, DMD Duchenne Muscular Dystrophy, SMA Spinal Muscular Atrophy, uNMD uncategorized NMD, SD standard deviation, FVC functional vital capacity, pp percent predicted, PCF peak cough flow, NIV non-invasive ventialation**,** LCI lung clearance index, N_2_ nitrogen, SF_6_ sulfur hexaflouride ^1^Two patients of this group did not perform this analysis ^2^One patient of this subgroup did not perform this analysis

Parameter	NMD	DMD	SMA	uNMD	Controls
n	24	12	8	4	15
Age [months] *mean (SD; range)*	198 (33.75; 121–239)	198.5 (15; 153–239)	170 (78.5; 121–220)	197.5 (28.25; 178–222)	205 (51; 105–224)
Female Sex [n (%)]	4 (16;7)	0 (0)	2 (25)	2 (50)	5 (33.3)
FVC [pp] *mean (SD; range)*	35.5 (19.25; 10–100)	40 (12; 24–100)	20 (9; 10–36)	36.5 (10.25; 22–48)	95 (18; 76–120)
PCF [L/min] *mean (SD; range)*	180 (145; 70–350)	235 (72.5; 140–350)	100 (15; 70–280)	160(70; 110–330)	470 (200; 180–750)
NIV n (%)	21 (87.5)	10 (83.3)	8 (100)	3 (75)	0 (0)
LCI N_2_ *mean (SD; range)*	9.67 (2.05; 7.66–13.27)	8.64 (1.14; 7.66–10.56)	11.36 (2.54; 7.73–13.27)	9.84 (1.45; 8.44–10.52)	7.03 (0.37; 6.32–7.43)
LCI SF_6_ pre *mean (SD;* *range)*	8.658.71 (1.52; 6.41–12.36)	8.09 (1.17; 6.41–9.54)	9.80 (2.65; 6.88–12.36)	8.20 (0.71; 6.69–9.20)	7.05 (0.67; 6.23–8.61)
LCI SF_6_ post *mean (SD;* *range)*	8.79 (2.03; 6.81–15.82)^1^	7.94 (1.11; 6.86–15.82)^2^	10.37 (2.57; 6.93–11.20)^2^	8.27 (0.51; 7.03–8.81)	na

## Discussion

In the present study both LCI N_2_ and LCI SF_6_ were higher in NMD patients (applying as well to every subgroup of NMD) as compared to healthy controls, indicating ventilation inhomogeneity in NMD patients with profound thoracic restriction. We demonstrate that lung involvement in NMD patients is more than muscular or thoracic restriction. The agreement of LCI N_2_ and LCI SF_6_ was good in NMD patients. Notably, we did not compare the subgroups of NMDs to one another, as the subgroups were too small and not matched. Elevated LCI in NMD patients correlated well to deterioration of FVC and PCF for both tracer gases used in our study. Ventilation inhomogeneity indicated by an elevated LCI SF_6_ with good correlation to FVC and PCF was shown before for DMD patients with profound thoracic restriction [5]. Our study now corroborates these findings for other groups of NMD as well. We show that the underlying ventilation inhomogeneity can be detected and monitored by both SF_6_ and N_2_ MBW. However, though both elevated, LCI N_2_ values were significantly higher in NMD patients than LCI SF_6_ values, which is in accordance to earlier observations in other disease cohorts but was not observed in our control group [15]. The reason for the relatively high difference between N_2_- and SF_6_-LCI values in our study cohort remains speculative. The different physical properties of both tracer gases might lead to distinct lung clearance especially in the context of altered ventilation mechanics in complex restrictive patterns of NMD patients. Speculatively, as hinted by higher LCI values for N_2_ in comparison to SF_6_, N_2_ MBW might be more sensitive towards detection of ventilation inhomogeneity in NMD patients. This might represent another reason to use N_2_ MBW in clinical practice in addition to environmental aspects regarding the greenhouse effects of SF_6_ [17]. We speculated that the use of cough assist devices might have an immediate influence on the observed ventilation inhomogeneity of NMD patients, for example by recruitment of (micro)atelectatic lung areas and/or mucus clearance. A moderate short-lasting increase of vital capacity could be demonstrated by the use of cough assists in NMD patients earlier [12]. However, despite a tendency towards lower values, LCI did not change significantly after the use of the cough assist. Though disappointing from a therapeutic perspective, this finding renders MBW lung function timing in NMD patients independent from cough assist use in future studies or in a clinical setting. Notably, the pressure levels of the cough assist devices used in this study can be regarded as low to moderate. Some centres use and recommend higher pressure levels (up to 60cmH_2_0), which might alter the influence on LCI [18]. Additionally, all but three out of 24 NMD patients used positive-pressure nocturnal non-invasive ventilation, which might decrease the effect of additional positive pressure on lung ventilation homogeneity delivered by cough assist devices. Future studies should investigate the effect of higher pressure levels in cough assist devices on the LCI, desirably in larger cohorts of NMD patients to allow comparison of NMD patient subgroups. Notably, the potential risk of pneumothorax through cough assist devices should be kept in mind, especially when increasing pressure levels [19].

In conclusion, NMD patients with respiratory muscle involvement develop lung ventilation inhomogeneity as a marker of secondary structural lung disease, which can be detected and monitored by N_2_ and SF_6_ MBW. This lung disease worsens with increasing muscular weakness. Mechanical assisted cough with low to moderate pressure levels does not have an immediate influence on lung ventilation inhomogeneity.

## Supplementary Information

**Additional file 1**. **Figure S1**. LCI N2 and SF6 plotted for the different NMDs of the study group and controls. LCI of all NMD subgroups differ significantly from the respective control group with the same tracer gas (*p* < 0.001). Mean and standard deviation are indicated. DMD Duchenne Muscular Dystrophy SMA Spinal Muscular Atrophy uNMD uncategorized neuromuscular disease.

## Data Availability

The datasets generated and/or analysed during the current study are available in the Figshare repository, https://figshare.com/s/942c1de31e11b9a26d63 (Private link. Public link will be generated after publishing of the paper.)

## Abbreviations

MBW: Multiple breath washout
LCI: Lung clearance index
NMD: Neuromuscular disease
DMD: Duchenne muscular dystrophy
SMA: Spinal muscular atrophy
NIV: Non-invasive ventilation
VC: Vital capacity
PCF: Peak cough flow
