# Supplementary material for: Multiple breath washout lung function reveals ventilation inhomogeneity unresponsive to mechanical assisted cough in patients with neuromuscular disease
Source: BMC Pulm Med. 2022 Jun 4;22:217. doi: 10.1186/s12890-022-02012-z (PMC9166427; doi:10.1186/s12890-022-02012-z)
Supplement: Supplementary file 1 — Additional file 1. Figure S1. LCI N2 and SF6 plotted for the different NMDs of the study group and controls. LCI of all NMD subgroups differ significantly from the respective control group with the same tracer gas (p < 0.001). Mean and standard deviation are indicated. DMD Duchenne Muscular Dystrophy SMA Spinal Muscular Atrophy uNMD uncategorized neuromuscular disease. [file 12890_2022_2012_MOESM1_ESM.docx]

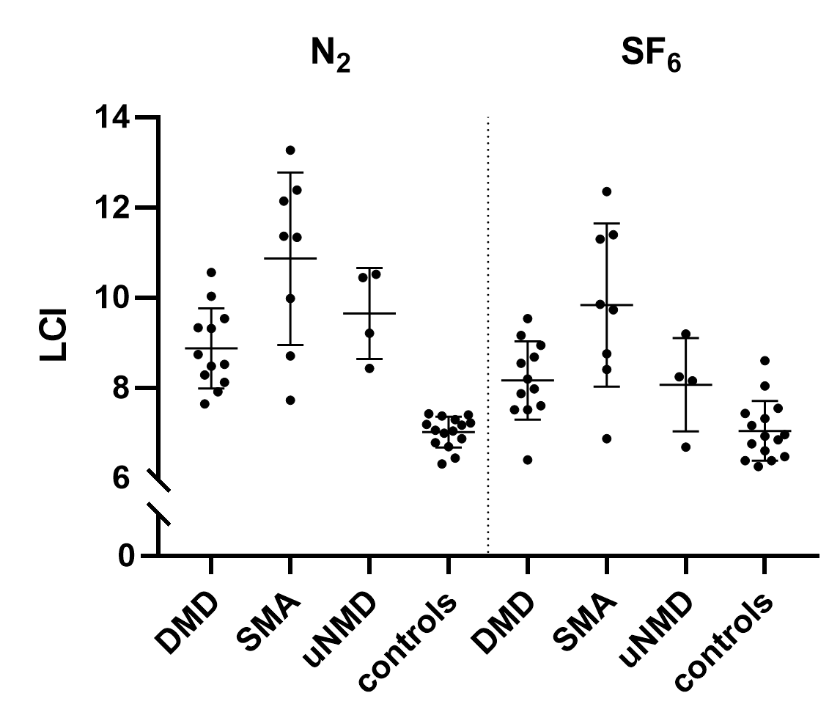


**Figure S1** LCI N_2_ and SF_6_ plotted for the different NMDs of the study group and controls. LCI of all NMD subgroups differ significantly from the respective control group with the same tracer gas (p<0.001). Mean and standard deviation are indicated. DMD Duchenne Muscular Dystrophy SMA Spinal Muscular Atrophy uNMD uncategorized neuromuscular disease
